# Sex difference in the associations among secondhand smoke with metabolic syndrome in non-smokers in a large Taiwanese population follow-up study

**DOI:** 10.7150/ijms.97306

**Published:** 2024-05-28

**Authors:** Tzu-Yu Chiang, Che-Sheng Pai, Jiun-Hung Geng, Pei-Yu Wu, Jiun-Chi Huang, Szu-Chia Chen, Jer-Ming Chang

**Affiliations:** 1 Department of post baccalaureate medicine, Kaohsiung Medical University, Kaohsiung, 807, Taiwan.; 2 Division of Endocrinology and Metabolism, Department of Internal Medicine, Kaohsiung Medical University Hospital, Kaohsiung Medical University, Kaohsiung, Taiwan.; 3 Department of Urology, Kaohsiung Municipal Siaogang Hospital, Kaohsiung Medical University, Kaohsiung, 812, Taiwan.; 4 Department of Urology, Kaohsiung Medical University Hospital, Kaohsiung Medical University, Kaohsiung, 807, Taiwan.; 5 Department of Internal Medicine, Kaohsiung Municipal Siaogang Hospital, Kaohsiung Medical University Hospital, Kaohsiung Medical University, Kaohsiung, 812, Taiwan.; 6 Division of Nephrology, Department of Internal Medicine, Kaohsiung Medical University Hospital, Kaohsiung Medical University, Kaohsiung, 807, Taiwan.; 7 Faculty of Medicine, College of Medicine, Kaohsiung Medical University, Kaohsiung, 807, Taiwan.; 8 Research Center for Precision Environmental Medicine, Kaohsiung Medical University, Kaohsiung, 807, Taiwan.

**Keywords:** sex difference, secondhand smoke, metabolic syndrome, Taiwan Biobank

## Abstract

Close associations among secondhand smoke (SHS) and metabolic syndrome (MetS) and its components have been demonstrated, however sex differences in these associations remain unclear. We collected 121,364 participants from the Taiwan Biobank, and excluded those with smoking history, the remaining 88,297 participants (male: 18,595; female: 69,702; mean age 50.1 ± 11.0 years) were included. SHS exposure was evaluated based on self-reported questionnaires. SHS was associated with MetS (odds ratio [OR], 1.268, *p* < 0.001 for males *vs.* 1.180, *p* < 0.001 for females), abdominal obesity (OR, 1.234, *p* < 0.001 for males *vs.* 1.199, *p* < 0.001 for females), low high-density lipoprotein cholesterol (OR, 1.183,* p* = 0.008 for males *vs.* 1.094,* p* = 0.011 for females), hyperglycemia (OR, 1.286,* p* < 0.001 for males *vs.* 1.234,* p* < 0.001 for females), but not with hypertriglyceridemia. SHS was associated with high blood pressure (BP) (OR, 1.278, *p* < 0.001) only in males, but not in females. Furthermore, significant interactions were found between sex x SHS on MetS (*p* = 0.023), abdominal obesity (*p* = 0.032), and elevated BP (*p* < 0.001). Moreover, the participants who were exposed to SHS for ≥1 hour per week were associated with a higher risk (OR = 1.316, *p* = 0.001 in males *vs.* OR = 1.220, *p* < 0.001 in females) of MetS compared to those with no exposure. These results showed an association between SHS and a high OR for MetS in both the males and females. Furthermore, sex differences were identified in the associations between SHS and MetS and its components, and SHS was more closely related to MetS, abdominal obesity, and high BP in males than in females.

## Introduction

Metabolic syndrome (MetS) was defined by National Cholesterol Education Program Adult Treatment Panel III guidelines 1 and modified criteria for Asians 2 as ≥3 of the following: (1) systolic/diastolic blood pressure (BP) ≥ 130/85 mmHg, a diagnosis of hypertension, or prescription for hypertensive medication; (2) triglyceride (TG) ≥ 150 mg/dL; (3) high-density lipoprotein cholesterol (HDL-C) < 50/40 mg/dL in women and men, respectively; (4) abdominal obesity (waist circumference [WC] ≥ 80/90 cm in women and men, respectively); (5) hyperglycemia (fasting whole-blood glucose concentration ≥ 100 mg/dL or a diagnosis of diabetes mellitus [DM]). The worldwide prevalence in adults has been estimated to range from 20-25% 3. Various factors and mechanisms have been associated with the etiology of MetS, including insulin resistance, dysfunction of adipose tissue, chronic inflammation, oxidative stress, disruption of the circadian rhythm, microbiota, genetic predisposition and maternal programming 4. Age, lifestyle factors and socioeconomic status also play major roles in the pathogenesis 5. MetS has been linked to the development of diseases including type 2 diabetes mellitus (DM), coronary diseases and stroke 6. Moreover, MetS-associated hyperglycemia, insulin and leptin resistance have been shown to have pro-inflammatory effects, which may lead to neuronal death and subsequently hormonal disbalance, cognitive decline, and increased immune sensitivity 7.

Exposure to secondhand smoke (SHS) is defined as currently being exposed to passive smoking indoors in the workplace or at home 8. Tobacco smoke comprises many components, of which 20 carcinogens, and in particular polycyclic aromatic hydrocarbons and the tobacco-specific nitrosamine 4-(methylnitrosamino)-1-(3-pyridyl)-1-butanone, are considered to play major roles in the development of lung cancer 9. The WHO estimates that nearly 7,000,000 deaths are directly attributable to the use of tobacco worldwide, and that approximately 1,200,000 non-smokers exposed to SHS die annually 10. Passive smoke is produced at different temperatures as well as different reducing conditions to active smoke, leading to even higher concentrations of some toxic substances in passive smoke 11. SHS exposure has also been linked to short sleep duration, poor sleep quality, delayed sleep onset, daytime sleepiness and parasomnias, particularly in children and those with pre-natal exposure 12. SHS has been associated with a higher overall cancer risk in never-smokers, particularly breast and lung cancers, and notably in females 13. A dose-response relationship has also been reported between coronary heart disease-related mortality in middle age and how many family members smoked during childhood 14. Furthermore, SHS exposure has been associated with an increased risk of chronic obstructive pulmonary disease (COPD), especially if the period of exposure is more than 5 years. In addition, SHS has been shown to particularly significantly increase the risk of developing COPD in those with a short duration of exposure and in females 15. Chen *et al.* identified a positive association between SHS and MetS and that this association varied with age, with younger people being more prone to lipid (low-density lipoprotein cholesterol [LDL-C] and HDL-C) metabolic disorders, and older people being more prone to glucose metabolic disorders. In addition, they showed that body mass index (BMI) was more obviously positively correlated in adults than in children and teenagers 16. Another study also supported an association between SHS exposure and MetS components in adolescents, with a higher urinary cotinine level being associated with a lower HDL-C level 17. Moreover, Kim *et al.* identified sex interactions in the association between SHS exposure and MetS. Their results indicated that female never-smokers had higher risks of all five MetS components if they had been exposed to SHS in comparison to those without exposure to SHS, while this was not found in male never-smokers 8.

Despite these findings, little research has been conducted on sex differences in the associations between SHS and MetS and its components. Therefore, we conducted this population-based study of over 120,000 participants in the Taiwan Biobank (TWB) to examine sex differences in these associations. In addition, we also examined sex differences in the association between the frequency of SHS exposure and MetS.

## Materials and methods

### Taiwan Biobank

To address the aging society in Taiwan with regards to health care promotion and chronic disease prevention, the TWB was established by the Ministry of Health and Welfare. The TWB enrolls cancer-free members of the community aged 30-70 years, and includes data on medical, genetic and lifestyle factors 18, 19. The Ethics and Governance Council of the TWB and Institutional Review Board (IRB) on Biomedical Science Research, Academia Sinica, Taiwan granted ethical approval for the TWB.

Data collected from all enrollees in the TWB include age, medical history (such as a diagnosis of DM and hypertension), body height/weight (BH/BW), hip circumferences (HC)/WC), and BMI. Eight-hour fasting serum samples were obtained from the participants for measurements of glucose, hemoglobin, TGs, LDL-C, HDL-C, total cholesterol, and uric acid (using a COBAS Integra 400, Roche Diagnostics GmbH, D-68298 Mannheim). Serum creatinine and estimated glomerular filtration rate (eGFR) were measured using the compensated Jaffé method and MDRD equation as reported previously 20.

Systolic and diastolic BPs measurements were also performed in each participant with an automated BP monitor by a trained staff member. All measurements were made in triplicate after abstaining from smoking, caffeine, and exercise for at least 30 minutes. We used average BP measurements for analysis. Regular exercise was defined according to the "Physical Fitness 333 Plan" of the Taiwan Ministry of Education: ≥ 30 minutes of physical activity for at least three times a week 21. The study protocol was approved by the IRB of Kaohsiung Medical University Hospital (KMUHIRB-E(I)-20210058), ensuring that it conformed to the ethical guidelines of the 1975 Declaration of Helsinki.

### Study participants

**Figure 1:**
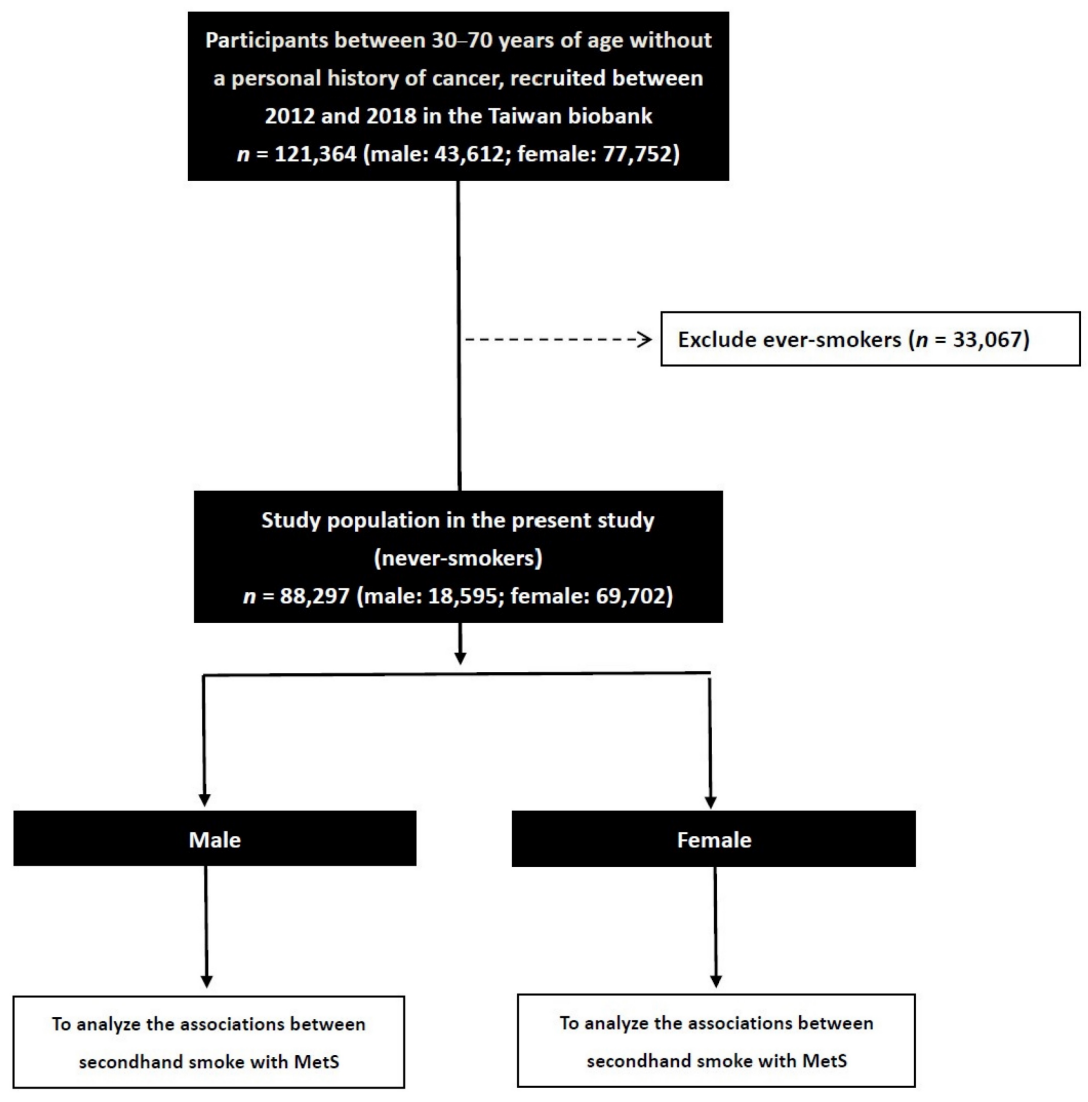
Flowchart of study population.

We collected 121,364 enrollees in the TWB (male: 43,612; female: 77,752), and excluded those with a smoking history (*n =* 33,067). The remaining 88,297 participants (male: 18,595; female: 69,702) were included to investigate sex differences in the associations between SHS and MetS (Figure 1).

### Definition of MetS

We used the National Cholesterol Education Program Adult Treatment Panel III guidelines 1 and modified criteria for Asians 2 to define MetS as ≥3 of the following: (1) systolic/diastolic BP ≥130/85 mmHg, a diagnosis of hypertension, or prescription for hypertensive medication; (2) TG ≥150 mg/dL; (3) HDL-C <50/40 mg/dL in women and men, respectively; (4) abdominal obesity (WC ≥80/90 cm in women and men, respectively); (5) hyperglycemia (fasting whole-blood glucose concentration ≥100 mg/dL or a diagnosis of DM).

### Smoking and SHS assessments

The participants completed self-reported questionnaires regarding smoking status, and they were then classified into “never-smoker”, “ex-smoker”, and “active-smoker” groups. We also asked the never-smokers “Have you been exposed to SHS?”. If the response was “No”, the participants were assigned to the non-exposure group, with the others being assigned to the exposure group, who were then also classified into “no exposure”, “<1 hour per week”, and “≥1 hour per week” groups based on their response to the question “How many hours per week have you been exposed to SHS?” We used 1 hour per week as the cutoff value as this was the median exposure time in this study.

### Menopause assessment

Female participants who did and did not report menopause after completing a questionnaire were classified the menopause and non-menopause groups, respectively.

### Statistical analysis

Statistical analysis was done using SPSS v19.0 (IBM Inc., Armonk, NY). Continuous variables were shown as mean ± SD, or medians (25th-75th percentiles) were used to describe the number of MetS, and comparisons were performed with the independent t test. Categorical variables were shown as frequency and percentage, and comparisons were performed with the chi-square test. Univariable and multivariable logistic regression analyses were performed to assess associations between SHS with MetS and its components. The second type included repeated sets of models including two-way interactions between each sex-related variable and SHS. An interaction *p* in logistic analysis: Model disease (y) = x1 + x2 + x1 × x2 + covariates. x1 × x2 was the interaction term, where y = MetS and its components; x1 = sex; x2 = SHS; covariates = age, regular exercise, history of alcohol and betel nut chewing, uric acid, hemoglobin, total cholesterol, eGFR and LDL-C. In order to ascertain the absence of potential outliers or influential data points that might influence the results, we performed residual analysis, encompassing the scrutiny of Cook's D and leverage values, to detect any outliers within the model. A Cook's D value less than 1 indicates no significant outliers in the regression model, while leverage values below 1 further reinforce the absence of outliers. Results were considered significant at a 2-tailed *p* value < 0.05.

## Results

The mean age of the 88,297 enrolled participants was 50.1 ± 11.0 years. Of the 18,595 male enrollees, 19.9% had MetS, compared to 22.6% of the 69,702 female enrollees (*p* < 0.001).

### Clinical characteristics of the male and female participants

The male participants were younger than the female participants. In addition, higher rates of hypertension, SHS exposure, alcohol/betel nut use, and regular exercise, and a lower rate of DM were noted in the males compared to the females. Moreover, the male participants had higher uric acid levels, systolic/diastolic BPs, WC/HC, BH/BW, BMI, LDL-C, fasting glucose, hemoglobin, and TGs, and lower eGFR, HDL-C and total cholesterol (Table 1). Furthermore, the male participants with MetS had more MetS components, a higher prevalence of MetS, hypertriglyceridemia and hyperglycemia and high BP, but lower rate of abdominal obesity and low HDL-C.

### Clinical characteristics of the males and females with and without MetS

The males with MetS were older than those without MetS. In addition, they had a higher prevalence of DM, hypertension, SHS exposure, alcohol and betel nut use, and lower frequency of regular exercise. Moreover, they had higher systolic/diastolic BPs, WC/HC, BH/BW, BMI, TGs, hemoglobin, fasting glucose, total cholesterol and uric acid, and lower eGFR and HDL-C (Table 2). Furthermore, the male MetS group had more components of MetS, and higher rates of all five components.

The female MetS group were older than those without MetS. In addition, they had higher rates of DM, hypertension, alcohol use, and regular exercise, and a lower prevalence rate of betel nut chewing. Moreover, they had higher systolic/diastolic BPs, WC/HC, BH/BW, BMI, uric acid, LDL-C, fasting glucose, total cholesterol, hemoglobin, and TGs, and lower eGFR and HDL-C (Table 2). Furthermore, the female MetS group had more components of MetS, and higher rates of all five components.

### Sex differences in the associations between SHS with MetS and its components

We performed multivariable logistic regression analysis to assess differences in sex-specific associations (Table 3). After adjusting for age, SHS, alcohol and betel nut use, regular exercise, hemoglobin, total cholesterol, LDL-C, eGFR and uric acid in the analysis of the male participants, SHS was associated with MetS (odds ratio [OR] = 1.268; 95% confidence interval [CI] = 1.125-1.428; *p* < 0.001), abdominal obesity (OR = 1.234; 95% CI = 1.112-1.371; *p* < 0.001), low HDL-C (OR = 1.183; 95% CI = 1.044-1.340; *p* = 0.008), hyperglycemia (OR = 1.286; 95% CI = 1.140-1.450; *p* < 0.001) and high BP (OR = 1.278; 95% CI = 1.151-1.418; *p* < 0.001), but not with hypertriglyceridemia (*p* = 0.103). In the female participants, SHS was associated with MetS (OR = 1.180; 95% CI = 1.093-1.275; *p* < 0.001), abdominal obesity (OR = 1.199; 95% CI = 1.127-1.275; *p* < 0.001), low HDL-C (OR = 1.094; 95% CI = 1.020-1.173; *p* = 0.011), and hyperglycemia (OR = 1.234; 95% CI = 1.139-1.338; *p* < 0.001), but not with hypertriglyceridemia (*p* = 0.106) or high BP (*p* = 0.058).

Furthermore, an interaction *p* in logistic analysis was performed: y = MetS and its components; x1 = sex; x2 = SHS. We found significant interactions between sex x SHS on MetS (β = 0.164; *p* = 0.023), abdominal obesity (β = 0.133; *p* = 0.032) and high BP (β = 0.278; *p* < 0.001). However, we did not find any significant differences in the other components (hypertriglyceridemia: β = 0.061, *p* = 0.407; low HDL-C: β = 0.125; *p* = 0.089; hyperglycemia: β = 0.046; *p* = 0.527). SHS was more strongly correlated with MetS, abdominal obesity and high BP in the male participants than in the female participants. Interaction between SHS with MetS and its components were seen more frequently in the female participants.

To ensure there are no indications of potential outliers or influential data points that may be driving the results, we conducted residual analysis, including examination of Cook's D and leverage values, to identify any outliers in the model. These analyses collectively indicate a well-fitting model without the presence of outliers, as Cook's D and leverage values less than 1.

### Sex differences in the association between the frequency of SHS exposure and MetS

Sex differences in the association between MetS and the frequency of SHS exposure were further explored in the three exposure groups (no exposure, <1 hour per week, and ≥1 hour per week) (Table 4). After adjusting for confounders, the male participants in the <1 hour per week exposure group (vs. no exposure; OR = 1.235; 95% CI = 1.037-1.471; *p* = 0.018), and ≥1 hour per week exposure group (vs. no exposure; OR = 1.316; 95% CI = 1.122-1.543; *p* = 0.001) were associated with a higher risk of MetS. In the female participants, those exposed to SHS ≥1 hour per week (vs. no exposure; OR = 1.220; 95% CI = 1.103-1.350; *p* < 0.001) were associated with a higher risk of MetS.

### Associations between SHS with MetS and its components in females by menopause status

Associations of SHS with MetS and its components in pre- and post-menopausal women were examined using multivariable logistic regression analysis (Table 5). After adjusting for age, alcohol and betel nut use, regular exercise, SHS, hemoglobin, total cholesterol, LDL-C, uric acid and eGFR, SHS was associated with MetS (OR = 1.260; 95% CI = 1.138-1.395; *p* < 0.001), abdominal obesity (OR = 1.342; 95% CI = 1.219-1.479; *p* < 0.001), hypertriglyceridemia (OR = 1.130; 95% CI = 1.0101.265; *p* = 0.034), low HDL-C (OR = 1.147; 95% CI = 1.034-1.272; *p* = 0.009), hyperglycemia (OR = 1.178; 95% CI = 1.063-1.305; *p* = 0.002) and high BP (OR = 1.155; 95% CI = 1.051-1.269; *p* = 0.003) in the post-menopausal women. In the pre-menopausal women, SHS was associated with abdominal obesity (OR = 1.123; 95% CI = 1.035-1.217; *p* = 0.005), and hyperglycemia (OR = 1.305; 95% CI = 1.147-1.485; *p* < 0.001), but not with MetS (*p* = 0.216), hypertriglyceridemia (*p* = 0.943), low HDL-C (*p* = 0.400) or high BP (*p* = 0.577).

Furthermore, an interaction *p* in logistic analysis was performed: y = MetS and its components; x1 = menopause; x2 = SHS. Significant interactions were found between menopause x SHS on abdominal obesity (β = -0.147; *p* = 0.022) and high BP (β = -0.173; *p* = 0.020). However, no significant differences were found in MetS (β = -0.132; *p* = 0.097), hypertriglyceridemia (β = -0.121; *p* = 0161), low HDL-C (β = -0.092; *p* = 0.198) and hyperglycemia (β = 0.094; *p* = 0.258). In the post-menopausal women, SHS was more strongly correlated with abdominal obesity and high BP than in the pre-menopausal women. Interaction between SHS with MetS components were seen more frequently in the post-menopausal women.

## Discussion

In our analysis of differences in the associations among SHS and MetS and its components between the male and female participants, the results showed that SHS was associated with a high OR for MetS in both groups. In addition, SHS was associated with high ORs for elevated BP, abdominal obesity, low HDL-C, and glucose impairment in males, and high ORs for abdominal obesity, low HDL-C, and glucose impairment in females. Moreover, we found sex differences in the relationships among SHS with MetS and the five components of MetS, and SHS was more closely related to MetS, abdominal obesity, and high BP in males than in females. Furthermore, the male and female participants with ≥1 hour per week of exposure to SHS were associated with 1.32- and 1.22-fold higher risks of MetS, respectively, compared to those with no exposure.

Our finding of an association between SHS and a high OR for MetS in both the male and female participants is important. A previous study 8 of 118,609 Korean participants over 18 years of age who self-reported that they were never-smokers and were verified through urinary cotinine analysis investigated the associations among SHS exposure, sex and prevalence of MetS, and reported a notably higher prevalence of MetS in both males and females exposed to SHS compared to those without exposure. In another large longitudinal study 22 of 71,055 non-smokers without MetS at baseline, the subjects were classified into continuous, former, new and no exposure to environmental tobacco smoke (ETS) groups. The results showed that new and continuous exposure to ETS led to a heightened risk of MetS compared to those without exposure (new exposure group, hazard ratio 1.35, *p* < 0.001; continuous exposure group, 1.19, *p* = 0.004). These results indicate that new exposure to ETS, even in individuals not previously exposed, raises the risk of MetS to a level similar to that for new exposure. In the present study, our results showed an association between SHS and MetS in both males and females, which is in line with the results of these studies. The impact of SHS on MetS may be due to several mechanisms 23. First, smoke-induced vascular changes can lead to a reduction in glucose uptake by skeletal muscles, and this may have a major effect on the progression of insulin resistance 23. Second, smoke has been shown to increase circulating white blood cell count, subsequently leading to carotid plaque formation, and such atherogenic consequences may partially explain the association with MetS 24. Although the pathophysiologic mechanisms underlying the development of SHS-induced MetS have yet to be fully elucidated, these proposed mechanisms may in part explain the correlation between SHS and MetS.

Our results further demonstrated that SHS was associated with a high OR for abdominal obesity in both males and females. In a cross-sectional analysis 25 of 4656 Korean men aged 19 to 79, the researchers used computed tomography to compare mean visceral adipose tissue between former and current smokers, and found that both groups were associated with increased visceral adipose tissue, in a manner proportional to the degree of exposure 25. Furthermore, another study demonstrated that cigarette smoke could increase gluteal adipose tissue lipoprotein lipase activity 26, upregulate the uptake and storage of TG fatty acids by adipocytes, thereby increasing fat mass 27. Our findings are in line with those of these studies, and may partially explain the possible mechanisms.

We also identified an association between SHS and a high OR for low HDL-C in both males and females. A study 28 of 1146 community-based male subjects in Taiwan aged ≥40 years found statistically significant dose-dependent associations between the amount of smoke exposure with MetS, high TG level, and low HDL-C level. Although the study excluded female subjects due to the low number of female smokers in Taiwan, their results support our findings, especially on the basis of ethnicity 28. Another study 25 involving 804 non-smoking adolescents from 1754 adolescents in the Western Australian Pregnancy cohort examined how passive smoking exposure impacted HDL-C levels, including the effects of smoking at home and during pregnancy. The findings revealed an association between decreased HDL-C levels and exposure to passive smoke from birth, and in particular in females aged 17 years (regression coefficient β = -0.09 [95% CI, -0.15 to -0.03]). The pathophysiologic impact of SHS exposure on low HDL-C may be explained as follows. Cigarette smoke may affect the critical enzymes involved in lipid transport 29, and a reduction in lecithin-cholesterol acyltransferase activity can impair HDL maturation leading to rapid clearance of nascent HDL from the circulation. Subsequently, changes in the activity of cholesterol ester transfer protein and hepatic lipase can both deplete cholesteryl ester and enrich TGs in HDL, and also decrease levels of HDL-C 29. Furthermore, an animal study also reported that the administration of nicotine resulted in increased levels of total cholesterol in rabbits and increased levels of plasma LDL in squirrel monkeys, and that the mechanism involved the increased breakdown of HDL and very low-density lipoprotein 30. In conclusion, the effect of cigarette smoking on reducing HDL-C levels may be through its impact on HDL metabolism.

Another interesting finding of the present study is the association between SHS exposure and a high OR for glucose impairment in the males and females. A previous meta-analysis study 16 suggested a positive association between SHS with fasting plasma glucose in people aged 27-74 years. Moreover, the Coronary Artery Risk Development in Young Adults (CARDIA) prospective study 11 demonstrated that both passive and active smoking were involved in developing glucose intolerance, with the highest 15-year incidence in smokers (21.8%), followed by non-smokers exposed to SHS (17.2%), former smokers (14.4%) and non-smokers not exposed to SHS (11.5%). Another study demonstrated that nicotine exposure could promote insulin resistance via the stimulation of transient extracellular signal-regulated kinase 1/2 and Ser636 and phosphorylation of insulin receptor substrate-1, and that this may be related to increased basal inhibition of insulin signaling 31. Furthermore, impaired β cell function is required for the development of glucose intolerance, and recent animal studies have demonstrated that prenatal nicotine exposure impairs pancreatic islet development through toxicity and apoptosis of β cell mitochondria 32, 33. Additionally, smoking has been shown to stimulate sympathetic neurotransmission through the effect of nicotine on stimulating the release of catecholamines 34. Catecholamines hinder insulin signaling, transduction pathways and the production of glucose transport proteins by increasing cyclic adenosine monophosphate (cAMP) levels 35. Both catecholamines and heightened cAMP levels can prompt a decrease in the number of insulin receptors in the plasma membranes of isolated fat cells. The biological associations among smoking, sympathetic neurotransmission and glucose impairment partially support our findings of an association between SHS exposure and glucose impairment.

The most important aspect of the present study was the investigation of sex differences in the associations between SHS and MetS and its components. Kim *et al.* 8 reported a dose-dependent association between SHS exposure and an especially higher prevalence of MetS in females who had never smoked. In addition, multivariate analysis revealed a significant association between exposure to SHS with MetS in both sexes, and that the risk was higher in the female participants (males: OR, 1.11 [95% CI, 1.04-1.20]; females: OR, 1.27 [95% CI, 1.17-1.37]). Specifically, females with ≥1 hour exposure per day and those exposed for ≥3 times a week had 22% and 30% higher risks of MetS, respectively (OR, 1.22 [95% CI, 1.02-1.45]; OR, 1.30 [95% CI, 1.14-1.49]). In contrast, we found opposite results in sex differences regarding the relationship between SHS and MetS compared to Kim *et al.*'s study 8, and we found that SHS was more closely related to MetS, abdominal obesity, and high BP in males than in females. Regarding abdominal obesity, our subgroup analyses showed that SHS was more strongly correlated to abdominal obesity in the post-menopausal participants than in the pre-menopausal participants. Rosano *et al.* 36 explored the possible intrinsic mechanisms, and found that estrogen withdrawal at menopause could trigger body fat distribution from a gynoid to an android pattern. This could then lead to predominant fat accumulation in the abdomen, thereby exacerbating abdominal obesity 36. These findings infer that estrogen has a protective effect in women between puberty and menopause, while this effect is not present in men, who are therefore more susceptible to abdominal obesity. Furthermore, a previous study 37 disclosed that estrogen favors the growth of subcutaneous adipose tissue over visceral adipose tissue in women as a starvation resistance mechanism, which then encourages the proliferation of adipocyte precursors in subcutaneous regions but the stimulation of lipolysis in abdominal regions. In contrast, the accumulation of meal-derived lipids is greater in visceral areas in men than in women, which may be related to androgen dihydrotestosterone 37. Sex differences also exist in the secretory function of adipocytes, such as higher leptin and adiponectin levels in women than in men 37. The aforementioned hormone-related mechanisms may explain why SHS has a greater impact in men through the effect of abdominal obesity than in women. Subgroup analyses of the female participants in our study revealed that SHS exposure was linked to elevated BP and abdominal obesity in the post-menopausal women, while such associations were not observed in the pre-menopausal women. These results also suggest the potential protective role of estrogen. Estrogen modulates BP both directly and indirectly. The direct effect is non-genomic and involves the reduction of calcium efflux in cardiac, renal and vascular cells, whereas the indirect effect is genomic and involves the inhibition of potent vasoconstrictors such as catecholamines, endothelin 1 and angiotensin II 38. However, testosterone was shown to have a pro-hypertensive effect by shifting renin-angiotensin-aldosterone system balance toward a pressor pathway in a rodent study, which found associations between testosterone with sodium retention in the kidneys, vascular and cardiac hypertrophy, and increased vasoconstriction 38. Moreover, Dai *et al.* identified that cigarette smoking was positively correlated with serum total and free testosterone concentrations 39, which further supports the higher testosterone level and higher BP in males exposed to SHS than in those not exposed to SHS. In summary, these findings suggest that the opposite impacts of different sex hormones on the cardiovascular system may partially explain why SHS was more closely related to high BP in the male participants than in the female participants in our study. Moreover, Flouris *et al.* suggested that sexual dimorphism in the effects of SHS may involve aspects other than sex hormones 40. The authors suggested that SHS exposure may be accompanied by notable elevations in the secretion of thyroid hormone and production of interleukin-1β in men, which then contributes to a more significant increase in systolic BP after SHS exposure in males compared with females. These studies partially verify our findings of the more positive correlation between SHS and high BP in males than in females.

Of note, differences between our study and Kim *et al.*'s study may explain the conflicting results 8. First, the definition of SHS exposure in our study was based on self-reported questionnaires, while the definition used in Kim *et al.*'s study was based on both self-reported questionnaires and urinary cotinine verification. However, urinary cotinine level may not completely represent SHS exposure due to sex differences in nicotine metabolic rate 41. Second, the ages of the study populations in the two studies were inconsistent, with an average age of about 35 years in Kim *et al.*'s study compared to about 50 years in ours, and in particular we included menopausal women. Different sex hormone status may therefore be a confounding factor. Third, the results may be affected by racial and genetic factors as well as underlying diseases in the two study populations.

This study is strengthened by the large number of participants to explore sex differences in the associations between SHS and MetS. Regarding the limitations, we could not identify how long the participants had had MetS as this was a cross-sectional study. Consequently, we could not examine causal relationships between SHS with MetS, and longitudinal studies are needed in the future to evaluate the risk of incident MetS. In addition, levels of the toxic chemicals found in SHS were not analyzed. Besides, BP measurements were also performed with an automated BP monitor. However, we could not obtain the information of the brand of automated BP monitors measured in each region. There may be some difference in terms of measuring BPs. Finally, the findings of this study may not be generalizable to other groups, as our participants were all ethnically Chinese.

In conclusion, the findings of this extensive cross-sectional study identified an association between SHS with a high OR for MetS in both men and women. Furthermore, we found sex differences in the relationships between SHS and MetS and its components, and SHS was more closely related to MetS, abdominal obesity, and high BP in males than in females. We hypothesize that these sex differences can be partially explained by hormone-related mechanisms, particularly estrogen and testosterone, as supported by our subgroup analysis results of stronger correlations between SHS and MetS and its components in post-menopausal women than in pre-menopausal women.

## Figures and Tables

**Table 1:** Clinical characteristics of the study participants classified by sex

Characteristics	Male (*n* = 18,595)	Female (*n* = 69,702)	*p*
Age (year)	48.7 ± 12.0	50.4 ± 10.7	< 0.001
DM (%)	4.3	5.5	< 0.001
Hypertension (%)	14.4	10.0	< 0.001
Secondhand smoking history (%)	9.7	7.4	< 0.001
Alcohol history (%)	7.6	1.7	< 0.001
Betel nut chewing history (%)	2.0	0.1	< 0.001
Regular exercise habits (%)	42.9	40.9	< 0.001
Systolic BP (mmHg)	125.8 ± 17.3	117.6 ± 18.7	< 0.001
Diastolic BP (mmHg)	78.1 ± 11.1	71.3 ± 10.7	< 0.001
Body height (cm)	169.8 ± 6.4	157.4 ± 5.6	< 0.001
Body weight (Kg)	72.5 ± 11.8	58.3 ± 9.7	< 0.001
Waist circumference (cm)	87.0 ± 9.3	80.6 ± 9.7	< 0.001
Hip circumference (cm)	97.5 ± 6.8	95.0 ± 7.1	< 0.001
BMI (kg/m^2^)	25.1 ± 3.5	23.5 ± 3.7	< 0.001
Laboratory parameters			
Fasting glucose (mg/dL)	97.8 ± 20.8	94.1 ± 18.6	< 0.001
Hemoglobin (g/dL)	15.0 ± 1.2	13.0 ± 1.3	< 0.001
Triglyceride (mg/dL)	125.3 ± 91.1	103.0 ± 74.1	< 0.001
Total cholesterol (mg/dL)	190.9 ± 34.2	198.0 ± 36.1	< 0.001
HDL-C (mg/dL)	48.8 ± 11.1	58.3 ± 13.2	< 0.001
LDL-C (mg/dL)	121.6 ± 30.9	120.6 ± 31.9	< 0.001
eGFR (mL/min/1.73 m^2^)	94.1 ± 19.2	108.4 ± 24.4	< 0.001
Uric acid (mg/dL)	6.4 ± 1.3	4.9 ± 1.1	< 0.001
MetS (%)	22.6	19.9	< 0.001
MetS numbers	2 (1-3)	1 (0-2)	< 0.001
MetS component			
Abdominal obesity (%)	35.4	50.1	< 0.001
Hypertriglyceridemia (%)	25.5	15.8	< 0.001
Low HDL-C (%)	19.4	27.1	< 0.001
Hyperglycemia (%)	24.5	17.1	< 0.001
High BP (%)	45.5	28.8	< 0.001

Abbreviations: DM, diabetes mellitus; BP, blood pressure; HDL-C, high-density lipoprotein cholesterol; LDL-C, low-density lipoprotein cholesterol; eGFR, estimated glomerular filtration rate; BMI, body mass index; MetS, metabolic syndrome.

**Table 2:** Clinical characteristics of the study participants classified by the presence of different sex and MetS

Characteristics		Male (*n* = 18,595)			Female (*n* = 69,702)		
	MetS (-) (*n* = 14,393)	MetS (+) (*n* = 4202)	*p*		MetS (-) (*n* = 55,800)	MetS (+) (*n* = 13,902)	*p*
Age (year)	48.0 ± 12.0	50.9 ± 11.5	< 0.001		49.2 ± 10.7	55.4 ± 9.3	< 0.001
DM (%)	3.5	12.5	< 0.001		1.8	14.6	< 0.001
Hypertension (%)	9.6	30.7	< 0.001		5.1	29.3	< 0.001
Secondhand smoke history (%)	9.3	11.3	< 0.001		7.4	7.7	0.224
Alcohol history (%)	7.0	9.9	< 0.001		1.8	1.5	0.040
Betel nut chewing history (%)	1.6	3.3	< 0.001		0.07	0.24	< 0.001
Regular exercise habits (%)	43.7	40.0	< 0.001		40.3	43.2	< 0.001
Systolic BP (mmHg)	122.9 ± 16.2	135.9 ± 17.0	< 0.001		113.9 ± 16.8	132.4 ± 18.6	< 0.001
Diastolic BP (mmHg)	76.2 ± 10.4	84.5 ± 11.0	< 0.001		69.6 ± 9.9	78.2 ± 10.9	< 0.001
Body height (cm)	169.8 ± 6.4	169.8 ± 6.4	< 0.001		157.6 ± 5.7	156.5 ± 5.6	< 0.001
Body weight (Kg)	70.0 ± 10.5	80.8 ± 12.4	< 0.001		56.6 ± 8.7	65.3 ± 10.5	< 0.001
Waist circumference (cm)	84.6 ± 8.2	95.0 ± 8.2	< 0.001		78.5 ± 8.7	89.0 ± 8.7	< 0.001
Hip circumference (cm)	96.2 ± 6.2	101.8 ± 7.0	< 0.001		94.1 ± 6.5	98.9 ± 7.8	< 0.001
BMI (kg/m^2^)	24.3 ± 3.1	28.0 ± 3.5	< 0.001		22.8 ± 3.2	26.6 ± 3.8	< 0.001
Laboratory parameters							
Fasting glucose (mg/dL)	94.7 ± 15.5	108.6 ± 30.8	< 0.001		90.6 ± 11.2	108.2 ± 31.4	< 0.001
Hemoglobin (g/dL)	15.0 ± 1.2	15.3 ± 1.2	< 0.001		12.9 ± 1.3	13.4 ± 1.3	< 0.001
Triglyceride (mg/dL)	103.1 ± 59.7	201.6 ± 130.5	< 0.001		84.6 ± 42.5	176.7 ± 116.0	< 0.001
Total cholesterol (mg/dL)	190.0 ± 33.2	193.8 ± 37.3	< 0.001		196.6 ± 35.3	203.6 ± 38.6	< 0.001
HDL-C (mg/dL)	51.2 ± 10.7	40.7 ± 8.1	< 0.001		61.1 ± 12.5	47.0 ± 9.4	< 0.001
LDL-C (mg/dL)	121.7 ± 30.3	121.3 ± 33.0	0.417		119.0 ± 31.2	126.9 ± 33.7	< 0.001
eGFR (mL/min/1.73 m^2^)	95.1 ± 18.7	90.6 ± 20.7	< 0.001		109.4 ± 24.0	104.0 ± 25.2	< 0.001
Uric acid (mg/dL)	6.2 ± 1.3	6.9 ± 1.5	< 0.001		4.7 ± 1.0	5.6 ± 1.2	< 0.001
MetS numbers	1 (0-2)	3 (3-4)	< 0.001		1 (0-2)	3 (3-4)	< 0.001
MetS component							
Abdominal obesity (%)	22.3	80.3	< 0.001		39.6	92.2	< 0.001
Hypertriglyceridemia (%)	12.7	69.4	< 0.001		5.2	58.0	< 0.001
Low HDL-C (%)	8.9	55.3	< 0.001		15.7	72.8	< 0.001
Hyperglycemia (%)	15.0	57.1	< 0.001		7.7	54.6	< 0.001
High BP (%)	34.9	81.8	< 0.001		18.3	70.8	< 0.001

Abbreviations are the same as in Table 1.

**Table 3:** Odds ratio for MetS and its components from secondhand smoke in different sex using multivariable logistic regression analysis

MetS and its components	Male (*n* = 18,595)				Female (*n* = 69,702)		
	Multivariable				Multivariable		
	OR	95% CI	*p*		OR	95% CI	*p*
Model 1: MetS	1.268	1.125-1.428	< 0.001		1.180	1.093-1.275	< 0.001
Model 2: Abdominal obesity	1.234	1.112-1.371	< 0.001		1.199	1.127-1.275	< 0.001
Model 3: Hypertriglyceridemia	1.107	0.980-1.251	0.103		1.072	0.985-1.166	0.106
Model 4: Low HDL-C	1.183	1.044-1.340	0.008		1.094	1.020-1.173	0.011
Model 5: Hyperglycemia	1.286	1.140-1.450	< 0.001		1.234	1.139-1.338	< 0.001
Model 6: High BP	1.278	1.151-1.418	< 0.001		1.072	0.998-1.151	0.058

Values expressed as odds ratio (OR) and 95% confidence interval (CI). Abbreviations are the same as in Table 1. Model 1: y = MetS, adjusted for x = age, secondhand smoke, alcohol and betel nut chewing history, regular exercise habits, hemoglobin, total cholesterol, LDL-C, eGFR and uric acid. Model 2: y = Abdominal obesity, adjusted for x = age, secondhand smoke, alcohol and betel nut chewing history, regular exercise habits, hemoglobin, total cholesterol, LDL-C, eGFR and uric acid. Model 3: y = Hypertriglyceridemia, adjusted for x = age, secondhand smoke, alcohol and betel nut chewing history, regular exercise habits, hemoglobin, total cholesterol, LDL-C, eGFR and uric acid. Model 4: y = Low HDL-C, adjusted for x = age, secondhand smoke, alcohol and betel nut chewing history, regular exercise habits, hemoglobin, total cholesterol, LDL-C, eGFR and uric acid. Model 5: y = Hyperglycemia, adjusted for x = age, secondhand smoke, alcohol and betel nut chewing history, regular exercise habits, hemoglobin, total cholesterol, LDL-C, eGFR and uric acid. Model 6: y = High BP, adjusted for x = age, secondhand smoke, alcohol and betel nut chewing history, regular exercise habits, hemoglobin, total cholesterol, LDL-C, eGFR and uric acid.

**Table 4:** Odds ratio for MetS according to frequency of secondhand smoke in different sex

Frequency of secondhand smoke	Multivariable	
	Odds ratio (95% CI)	*p*
Male		
No exposure	Reference	
< 1 hour per week exposure	1.235 (1.037-1.471)	0.018
≥ 1 h per week exposure	1.316 (1.122-1.543)	0.001
Female		
No exposure	Reference	
< 1 hour per week exposure	1.104 (0.975-1.250)	0.117
≥ 1 h per week exposure	1.220(1.103-1.350)	< 0.001

Values expressed as odds ratio and 95% confidence interval (CI). Abbreviations are the same as in Table 1. Adjusted for age, secondhand smoke, alcohol and betel nut chewing history, regular exercise habits, hemoglobin, total cholesterol, LDL-C, eGFR and uric acid.

**Table 5:** Odds ratio for MetS and its components from secondhand smoke in females according to menopause using multivariable logistic regression analysis

MetS and its components	Female menopause (+) (*n* = 35,764)				Female menopause (-) (*n* = 33,911)		
	Multivariable				Multivariable		
	OR	95% CI	*p*		OR	95% CI	*p*
Model 1: MetS	1.260	1.138-1.395	< 0.001		1.079	0.956-1.218	0.216
Model 2: Abdominal obesity	1.342	1.219-1.479	< 0.001		1.123	1.035-1.217	0.005
Model 3: Hypertriglyceridemia	1.130	1.010-1.265	0.034		0.995	0.875-1.132	0.943
Model 4: Low HDL-C	1.147	1.034-1.272	0.009		1.043	0.946-1.151	0.400
Model 5: Hyperglycemia	1.178	1.063-1.305	0.002		1.305	1.147-1.485	< 0.001
Model 6: High BP	1.155	1.051-1.269	0.003		0.968	0.865-1.084	0.577

Values expressed as odds ratio (OR) and 95% confidence interval (CI). Abbreviations are the same as in Table 1. Model 1: y = MetS, adjusted for x = age, secondhand smoke, alcohol and betel nut chewing history, regular exercise habits, hemoglobin, total cholesterol, LDL-C, eGFR and uric acid. Model 2: y = Abdominal obesity, adjusted for x = age, secondhand smoke, alcohol and betel nut chewing history, regular exercise habits, hemoglobin, total cholesterol, LDL-C, eGFR and uric acid. Model 3: y = Hypertriglyceridemia, adjusted for x = age, secondhand smoke, alcohol and betel nut chewing history, regular exercise habits, hemoglobin, total cholesterol, LDL-C, eGFR and uric acid. Model 4: y = Low HDL-C, adjusted for x = age, secondhand smoke, alcohol and betel nut chewing history, regular exercise habits, hemoglobin, total cholesterol, LDL-C, eGFR and uric acid. Model 5: y = Hyperglycemia, adjusted for x = age, secondhand smoke, alcohol and betel nut chewing history, regular exercise habits, hemoglobin, total cholesterol, LDL-C, eGFR and uric acid. Model 6: y = High BP, adjusted for x = age, secondhand smoke, alcohol and betel nut chewing history, regular exercise habits, hemoglobin, total cholesterol, LDL-C, eGFR and uric acid.
